# EMPOWERING SENIORS TO RECOGNIZE ELDER MISTREATMENT THROUGH PEER SUPPORT: RESULTS FROM A COMMUNITY OUTREACH PROJECT

**DOI:** 10.1093/geroni/igac059.2290

**Published:** 2022-12-20

**Authors:** Jessica Hsieh, Sharon Tan, Celeste Pang, Raza Mirza, Christopher Klinger

**Affiliations:** University of Toronto, Toronto, Ontario, Canada; University of Toronto, Toronto, Ontario, Canada; Egale, Toronto, Ontario, Canada; National Initiative for the Care of the Elderly, Toronto, Ontario, Canada; University of Toronto, Toronto, Ontario, Canada

## Abstract

Elder mistreatment, often understood in the context of abuse and neglect, is a growing concern for the health and wellbeing of seniors and their families. A 2015 Canadian prevalence study by the National Initiative for the Care of the Elderly (NICE) found that seniors who are mistreated are more willing to talk to other seniors, rather than clinicians, police or family. However, a lack of knowledge, access to resources, and community stigma may limit seniors’ abilities to address mistreatment.This study evaluated the impact on knowledge, attitudes, and behaviours of having seniors deliver workshops on mistreatment to other seniors. A Seniors’ Advisory Committee developed content for these workshops, delivered by seniors in sixteen communities across Ontario, Canada. Participants completed pre/post-surveys assessing changes in knowledge, attitudes, and behaviours. Results indicate that workshops effectively increased awareness of mistreatment issues among participants, on average, by 37%. Participants experienced a 44% increase in their perceived preparation to provide information to another older adult asking about mistreatment. Barriers to help-seeking among seniors include finding trustworthy sources, fear of retaliation, and a lack of legal protection. Prior to the training, healthcare providers were the main sources of information for participants (58%); depending on the severity of the situation, 60% of participants indicated eventually reporting to police, who are not the preferred source of information. Senior-led workshops about mistreatment appear to be effective for increasing knowledge and encouraging disclosure and help-seeking behaviours. Results support prevention models that empower seniors to educate other seniors on issues around mistreatment.

